# Lapatinib as first-line treatment for muscle-invasive urothelial carcinoma in dogs

**DOI:** 10.1038/s41598-021-04229-0

**Published:** 2022-01-13

**Authors:** Shingo Maeda, Kosei Sakai, Kenjiro Kaji, Aki Iio, Maho Nakazawa, Tomoki Motegi, Tomohiro Yonezawa, Yasuyuki Momoi

**Affiliations:** 1 Department of Veterinary Clinical Pathobiology, Graduate School of Agricultural and Life Sciences, The University of Tokyo, 1-1-1, Yayoi, Bunkyo-ku, Tokyo, 113-8657 Japan; 2 Veterinary Medical Center, Graduate School of Life and Environmental Sciences, Osaka Prefecture University, Osaka, Japan; 3 Veterinary Medical Center, The University of Tokyo, Tokyo, Japan

**Keywords:** Cancer models, Cancer therapy, Urological cancer

## Abstract

Epidermal growth factor receptors 1 and 2 (EGFR and HER2) are frequently overexpressed in various malignancies. Lapatinib is a dual tyrosine kinase inhibitor that inhibits both EGFR and HER2. Although a phase III trial failed to show the survival benefits of lapatinib treatment after first-line chemotherapy in patients with EGFR/HER2-positive metastatic urothelial carcinoma, the efficacy of lapatinib for untreated urothelial carcinoma is not well defined. Here, we describe the therapeutic efficacy of lapatinib as a first-line treatment in a canine model of muscle-invasive urothelial carcinoma. In this non-randomized clinical trial, we compared 44 dogs with naturally occurring urothelial carcinoma who received lapatinib and piroxicam, with 42 age-, sex-, and tumor stage-matched dogs that received piroxicam alone. Compared to the dogs treated with piroxicam alone, those administered the lapatinib/piroxicam treatment had a greater reduction in the size of the primary tumor and improved survival. Exploratory analyses showed that HER2 overexpression was associated with response and survival in dogs treated with lapatinib. Our study suggests that lapatinib showed encouraging durable response rates, survival, and tolerability, supporting its therapeutic use for untreated advanced urothelial carcinoma in dogs. The use of lapatinib as a first-line treatment may be investigated further in human patients with urothelial carcinoma.

## Introduction

Bladder cancer, also known as urothelial carcinoma, is a prevalent and aggressive malignancy, with an estimated 0.6 million new cancer cases and 199,900 deaths annually worldwide^1^. This cancer is histologically classified into two types: low-grade and high-grade. While the low-grade, superficial type, is more prevalent (~ 70%) and related to a favorable prognosis, the high-grade muscle-invasive urothelial carcinoma is less prevalent (~ 30%) with a high mortality risk due to metastasis^2^. Platinum-based chemotherapy, a first-line standard of care for advanced disease, provides clinical benefit; however, the prognosis is poor, with an overall survival of approximately 9–15 months^3,4^. The 5-year survival rate is approximately 5% in metastatic urothelial carcinoma. Moreover, up to two-thirds of patients are ineligible for cisplatin-based chemotherapy due to substantial side effects or comorbidities (e.g., renal dysfunction)^5^. New approaches as a first-line treatment are warranted to break these therapeutic stalemates.

Epidermal growth factor receptors 1 and 2 (EGFR and HER2) are frequently overexpressed in subsets of various solid tumors. Heterodimerization of these receptors results in the autophosphorylation of tyrosine residues within the cytoplasmic domain of the heterodimer and initiates signaling pathways leading to cellular proliferation and carcinogenesis^6^. Lapatinib, a dual inhibitor of EGFR and HER2 tyrosine kinases, is an established therapeutic drug in breast cancer harboring HER2 overexpression and/or gene amplification^7^. EGFR and HER2 have also been implicated in bladder cancer progression^8^. Although preclinical and phase II trial data support the use of lapatinib in urothelial carcinoma^9,10^, a phase III randomized controlled trial failed to show survival benefits of lapatinib treatment after first-line chemotherapy in patients with EGFR/HER2-positive metastatic urothelial carcinoma^11^. However, the clinical efficacy of lapatinib as first-line treatment for untreated urothelial carcinoma remains unclear.

Naturally occurring urothelial carcinoma in dogs resembles human bladder cancer in terms of genetic and environmental heterogeneity, clinical signs, histopathology, disease progression, metastatic behavior, and response to cisplatin-based chemotherapy^12–14^. Unlike humans, the high-grade muscle-invasive type is the leading canine urothelial carcinoma (> 90% of cases), whereas the low-grade superficial type is infrequent in dogs. Studies using microarray and RNA sequencing (RNA-Seq) have shown similarities in gene expression profiles between canine and human urothelial carcinomas^15–17^. Furthermore, dog-based clinical trials can be conducted in a comparatively short duration because dogs have a shorter life span than humans. Thus, the canine urothelial carcinoma model could be informative for evaluating therapeutics for muscle-invasive bladder cancer.

Overexpression of EGFR and HER2 is observed in approximately 70% and 60% of canine urothelial carcinoma cases, respectively^18,19^. This is comparable to or higher than the rates found in human bladder cancer^20–22^. Our previous RNA-Seq study showed that HER2 was the most activated upstream regulator related to carcinogenesis in dogs with urothelial carcinoma^16^. In addition, we showed the antitumor effect of lapatinib in canine urothelial carcinoma cell lines in vitro and in a xenograft mouse model^23^. As the first prospective non-randomized clinical trial, the present study aimed to evaluate the therapeutic potential of lapatinib as a first-line treatment in dogs with spontaneous muscle-invasive urothelial carcinoma. We also evaluated whether HER2 overexpression/amplification predicted the clinical efficacy of lapatinib treatment.

## Results

### Case population

Ninety-one dogs were screened for inclusion in the study between August 2017 and September 2019 at the Veterinary Medical Center of the University of Tokyo (VMC-UT). Of these, five dogs were excluded because of incomplete enrollment information, hydronephrosis caused by tumor obstruction, or end-stage urothelial carcinoma. A total of 86 dogs with urothelial carcinoma were enrolled in this non-randomized clinical trial. Overall, case dogs included 59 females (11 intact and 48 spayed) and 27 males (9 intact and 18 castrated), and the median age was 11.9 years (range 6.3–16.2 years). Based on the World Health Organization (WHO) TNM classification for canine bladder cancer^24,25^, 77 of 86 (90%) tumors were classified as T2 (muscle-invasive) and 9 of 86 (10%) as T3 (tumor invading neighboring organs). No cases were classified as T1 (superficial papillary tumors). We detected nodal and distant (to the lung) metastases in 24 (28%) and 5 (6%) dogs, respectively. Forty-four of these dogs were assigned to the lapatinib/piroxicam arm, and the remainder (*n* = 42) were assigned to the piroxicam arm according to the dog owners’ preferences. In lapatinib/piroxicam arm, 32/44 dogs had received nonsteroidal anti-inflammatory drugs (NSAIDs) prior to lapatinib treatment including firocoxib, carprofen, or piroxicam for a median of 112 days. In piroxicam arm, 27/42 dogs had received NSAIDs prior to the trial including, firocoxib, carprofen, meloxicam, or piroxicam for a median of 98 days. All NSAIDs administered at the start of the clinical trial were unified to piroxicam. The baseline characteristics of the dogs used in this clinical trial are summarized in Table 1 and Table S1. There were no significant differences in clinical variables, including age, body weight, gender, tumor characteristics, metastasis, and BRAF gene status, between the treatment groups (Table 1).

**Table 1:** Baseline subject characteristics in the clinical trial.

Characteristics	Lapatinib/piroxicam (*n* = 44)	Piroxicam (*n* = 42)	*P*
	No. of dogs (%)	No. of dogs (%)	
**Age**^a^** (years)**			
Median	12.3	11.9	0.44
Range	6.3–16.2	7–15.9	
**Body weight**^a^** (kg)**			
Median	5.5	6.5	0.09
Range	2.5–36.1	1.3–37.6	
**Gender**^b^			
Female neutered	27 (61.3)	21 (50.0)	0.79
Female intact	5 (11.4)	6 (14.3)	
Male neutered	8 (18.2)	10 (23.8)	
Male intact	4 (9.1)	5 (11.9)	
Muscle-invasive tumor^b^ (T2 or T3)	44 (100)	42 (100)	1.00
**Tumor volume**^a^** (mm**^**3**^**)**			
Median	2872.1	2835.7	0.62
Range	338.2–18,299.9	216.7–29,751.9	
**Tumor location**^b^			
Vesical apex	7 (15.9)	6 (14.3)	0.39
Vesical body	15 (34.1)	12 (28.6)	
Vesical trigone	22 (50.0)	22 (52.4)	
Urethra involvement	11 (25.0)	12 (28.6)	
Prostate involvement	3 (6.8)	2 (4.8)	
**Metastasis**^b^			
Any	13 (29.5)	13 (31.0)	1.00
Lymph node	13 (29.5)	11 (26.2)	
Lung	2 (4.5)	3 (7.1)	
**BRAF gene mutation**^b^			
Wild-type	12 (27.3)	11 (26.2)	1.00
BRAF^V595E^ mutation	32 (72.7)	31 (73.8)	

^a^The Mann–Whitney *U* test.

^b^Fisher’s exact test.

### Clinical responses, outcomes, and adverse events

**Figure 1:**
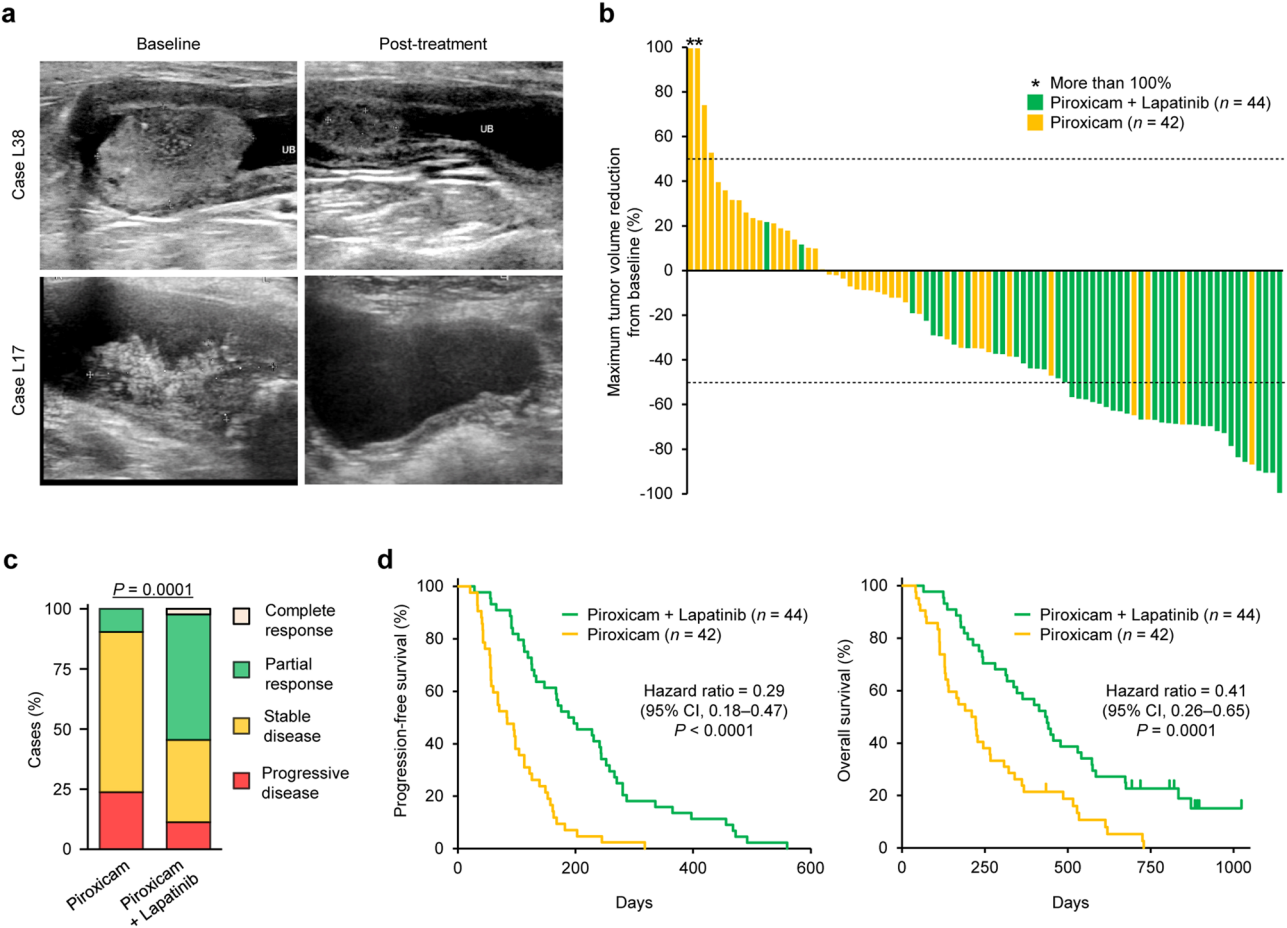
Lapatinib induces clinical responses and improves the survival in dogs with urothelial carcinoma. (**a**) Representative ultrasonographic images of bladder masses in dogs treated with lapatinib and piroxicam. In case L38, the bladder mass shrunk 4 weeks after treatment compared to baseline. In case L17, the bladder mass observed before treatment was completely absent 12 weeks after treatment. (**b**) Waterfall plot showing the maximum percentage of tumor burden reduction from baseline in dogs treated with lapatinib and piroxicam (*n* = 44, green) or in dogs treated with piroxicam alone (*n* = 42, yellow). Dashed lines indicate ± 50%. Asterisk indicates a value > 100%. (**c**) Clinical responses in dogs treated with lapatinib and piroxicam (*n* = 44) or piroxicam alone (*n* = 42). Cochran–Armitage test. (**d**) Progression-free survival (left) and overall survival (right) in dogs treated with lapatinib and piroxicam (*n* = 44, green) or in dogs treated with piroxicam alone (*n* = 42, yellow). Log-rank test and Cox proportional hazard model.

To evaluate the clinical activity of lapatinib treatment in spontaneous canine muscle-invasive urothelial carcinoma, we compared 44 dogs in the lapatinib/piroxicam arm with 42 dogs in the piroxicam arm. Most dogs that received lapatinib in combination with piroxicam showed a reduction in tumor burden (Fig. 1a,b). Among the 44 dogs with lapatinib/piroxicam, 1 (2%) had a complete remission (CR), 23 (52%) had a partial response (PR), 15 (34%) had stable disease (SD), and 5 (12%) had progressive disease (PD). In 42 dogs with piroxicam alone, 4 (9%) had PR, 28 (67%) had SD, and 10 (24%) had PD. The clinical responses in dogs treated with lapatinib in combination with piroxicam were superior to those with piroxicam alone (Fig. 1c). At the cutoff time for the study data (July 1, 2021), 8 (18%) dogs that received lapatinib treatment and 1 (2%) dog with piroxicam alone were still alive. The median progression-free survival (PFS) in dogs treated with lapatinib/piroxicam and dogs treated with piroxicam alone were 193 (range 28–560) days and 90 (range 21–318) days, respectively (hazard ratio 0.29; 95% CI 0.18–0.47; *P* < 0.0001). The median overall survival (OS) in dogs treated with lapatinib/piroxicam and dogs treated with piroxicam alone were 435 (range 65–1023) days and 216 (range 41–725) days, respectively (hazard ratio 0.41; 95% CI 0.26–0.65; *P* = 0.0001). The PFS and OS in dogs treated with lapatinib in combination with piroxicam was longer than that in dogs treated with piroxicam alone (Fig. 1d).

The median duration of treatment was 221 (range 55–1023) days for dogs with lapatinib/piroxicam and 128 (range 33–703) days for dogs with piroxicam alone. Lapatinib/piroxicam and piroxicam treatment continued for over 6 months in 24/44 (55%) dogs and 16/42 (38%) dogs, respectively. Although dogs with lapatinib/piroxicam had a greater opportunity to experience adverse safety changes due to the longer time on treatment compared to dogs with piroxicam alone, no adjustments were made in the statistical comparisons for this disparity. Of the 44 dogs with lapatinib/piroxicam, 36 (82%) had an adverse event (Table 2). Most treatment-related adverse events were grade 1 or 2, and many were transient. The leading adverse events were increased alkaline phosphatase (ALP; 48%), increased alanine aminotransferase (ALT; 45%), vomiting (18%), diarrhea (18%), anorexia (11%), increased total bilirubin (11%), and increased creatinine (11%). There were no grade 4–5 treatment-related adverse events. Although no dog exhibited an event leading to discontinuation, the lapatinib dose was reduced in four dogs (9%). There were no cases of delayed administration. We observed dermatologic adverse events in 5 cases (11%), including hyperpigmentation, pruritus, skin ulceration, and alopecia. Grade 3 pulmonary hypertension was detected in one dog (2%), which was treated with sildenafil. Of the 42 dogs treated with piroxicam alone, 17 (40%) had an adverse event (Table 2). The leading adverse events were increased creatinine (17%), anorexia (14%), increased ALP (12%), and vomiting (10%). The incidence of overall adverse events, increased ALT, and increased ALP was significantly higher in dogs with lapatinib/piroxicam than in dogs with piroxicam alone, but there were no significant differences in other adverse events between the treatment groups (Table 2).

**Table 2:** Adverse events in dogs treated with lapatinib/piroxicam or piroxicam alone.

Event^a^	Number of cases (%)								*P*^c^
	Lapatinib/piroxicam (*n* = 44)				Piroxicam (*n* = 42)				
	Any grade	Grade 1	Grade 2	Grade 3	Any grade	Grade 1	Grade 2	Grade 3	
Any event	36 (81.8)	33 (75.0)	13 (29.5)	1 (2.3)	17 (40.5)	13 (31.0)	3 (7.1)	0	< 0.01
**Gastrointestinal**									
Vomiting	8 (18.2)	6 (13.6)	2 (4.5)	0	4 (9.5)	3 (7.1)	1 (2.4)	0	0.35
Diarrhea	8 (18.2)	5 (11.4)	3 (6.8)	0	2 (4.8)	2 (4.8)	0	0	0.09
Anorexia	5 (11.4)	2 (4.5)	3 (6.8)	0	6 (14.3)	4 (9.5)	2 (4.8)	0	0.75
Stomatitis	1 (2.3)	0	1 (2.3)	0	0	0	0	0	1.00
**Dermatologic**									
Hyperpigmentation	3 (6.8)	1 (2.3)	2 (4.5)	0	0	0	0	0	0.24
Pruritus	1 (2.3)	0	1 (2.3)	0	0	0	0	0	1.00
Skin ulceration	1 (2.3)	0	1 (2.3)	0	0	0	0	0	1.00
Alopecia	1 (2.3)	1 (2.3)	0	0	0	0	0	0	1.00
**Cardiac**									
Pulmonary hypertension	1 (2.3)	0	0	1 (2.3)	0	0	0	0	1.00
**Metabolic**^b^									
Increased ALT	20 (45.5)	15 (34.1)	5 (11.4)	0	2 (4.8)	2 (4.8)	0	0	< 0.01
Increased ALP	21 (47.7)	13 (29.5)	8 (18.2)	0	5 (11.9)	4 (9.5)	1 (2.4)	0	< 0.01
Increased total bilirubin	5 (11.4)	5 (11.4)	0	0	0	0	0	0	0.06
Increased creatinine	5 (11.4)	5 (11.4)	0	0	7 (16.7)	6 (14.3)	1 (2.4)	0	0.54

^a^Toxicity grade based on published criteria^48^.

^b^*ALT* alanine aminotransferase, *ALP* alkaline phosphatase.

^c^Fisher’s exact test.

Overall, these results suggest that lapatinib treatment leads to clinical responses and improves survival in dogs with muscle-invasive urothelial carcinoma. The incidence of hepatotoxic adverse events was greater in the lapatinib/piroxicam combination treatment than the piroxicam alone, but the results may not result in more clinically significant adverse events.

### Biomarker assessments

**Figure 2:**
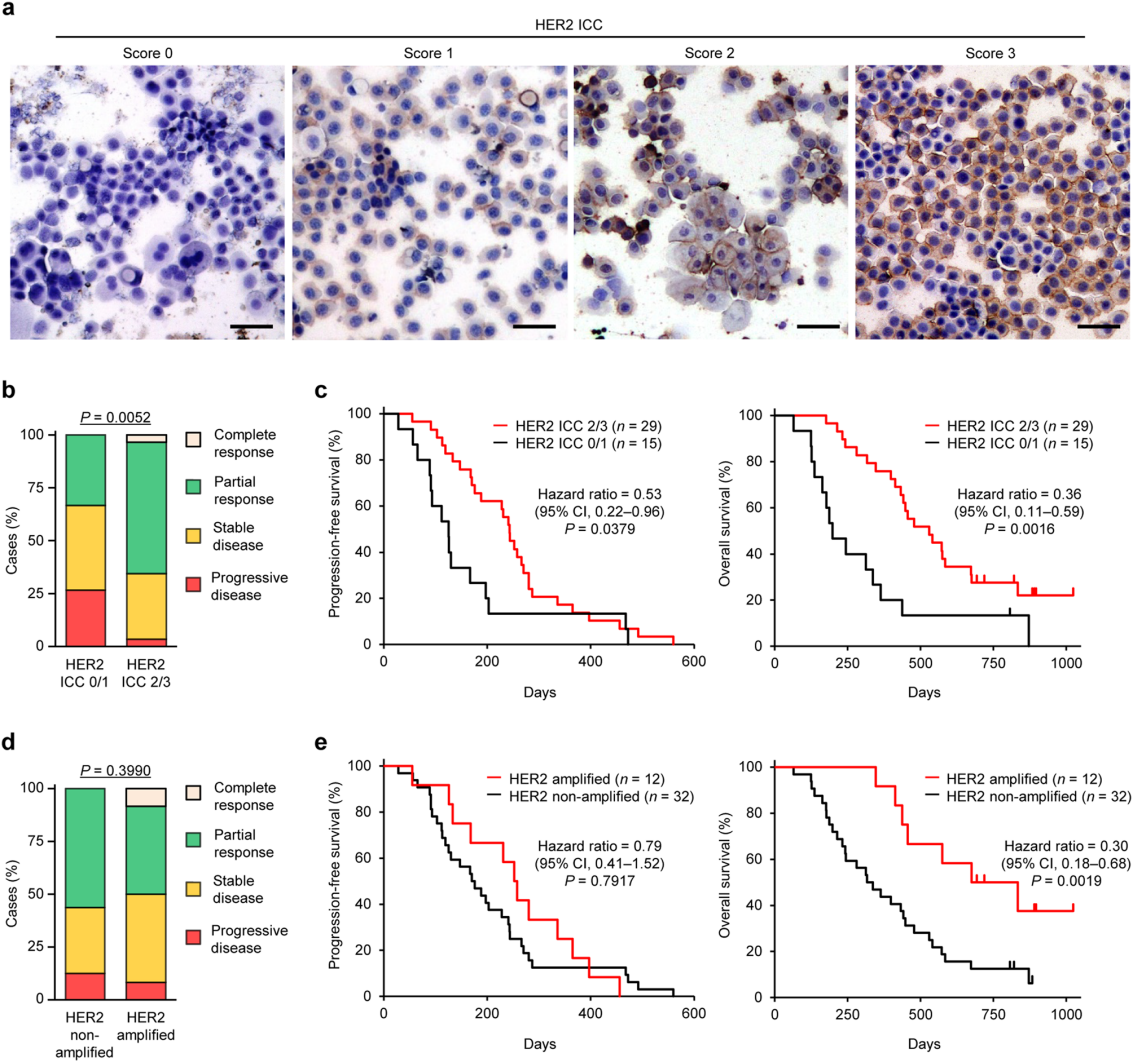
Human epidermal growth factor receptor-2 (HER2) status is associated with the clinical responses and outcomes of lapatinib treatment. (**a**) Representative images of immunocytochemistry (ICC) for HER2 in canine urothelial carcinoma. A score of 0 denotes no reactivity, a score of 1 represents incomplete and weak immunoreactivity in < 10% of tumor cells, a score of 2 represents incomplete but intense immunoreactivity in < 10% of tumor cells, and a score of 3 represents intense with complete immunoreactivity in ≥ 10% of tumor cells. Samples with HER2 ICC scores of 0 and 1 were classified as negative, and those with scores of 2 and 3 as positive. Scale bars, 50 μm. (**b**) Association of HER2 overexpression with response to treatment in dogs treated with lapatinib and piroxicam (*n* = 44). Cases were classified as either HER2 positive (ICC 2/3; *n* = 29) or HER2 negative (ICC 0/1; *n* = 15). Cochran–Armitage test. (**c**) Kaplan–Meier curves of progression-free survival (left) and overall survival (right) according to HER2 overexpression in dogs treated with lapatinib and piroxicam (*n* = 44). Log-rank test and Cox proportional hazard model. (**d**) Association of *HER2* gene amplification with response to treatment in dogs treated with lapatinib and piroxicam (*n* = 44). Cases were classified as HER2 amplified (*n* = 12) or HER2 non-amplified (*n* = 32). Cochran–Armitage test. (**e**) Kaplan–Meier curves of progression-free survival (left) and overall survival (right) according to HER2 gene amplification in dogs treated with lapatinib and piroxicam (*n* = 44). Log-rank test and Cox proportional hazard model.

We have previously shown that the antitumor effect of lapatinib in canine urothelial carcinoma cell lines is associated with HER2 expression, but not with EGFR expression^23^. Thus, we examined the association between HER2 status and response to lapatinib treatment in this canine clinical trial. In 44 dogs that received lapatinib treatment, HER2 expression was examined in 19 dogs by immunohistochemistry (IHC) and all dogs by immunocytochemistry (ICC) (Table S1). HER2 positivity examined by ICC was associated with that determined by IHC (Table 3). HER2 positivity (HER2 ICC 2/3) was observed in 29 of 44 (66%) cases (Fig. 2a) and was associated with a favorable response in dogs treated with lapatinib/piroxicam (Fig. 2b). Similarly, PFS and OS for HER2 positive cases were longer than those for HER2 negative cases (Fig. 2c). HER2 gene amplification was detected in 12 of 44 (27%) dogs treated with lapatinib/piroxicam (Table S1). In 29 HER2 positive cases, HER2 gene amplification was observed in 11 dogs (38%). HER2 gene amplification was associated with HER2 positivity (Table 4), but not with clinical response in dogs treated with lapatinib/piroxicam (Fig. 2d). The OS for cases with HER2 gene amplification was longer than that for cases without HER2 amplification (Fig. 2e).

**Table 3:** Comparison of HER2 immunocytochemistry and immunohistochemistry in dogs with urothelial carcinoma.

HER2 immunohistochemistry	HER2 immunocytochemistry			*P*^a^
	ICC 2/3	ICC 0/1	Total	
IHC 2/3	14	2	16	0.01
IHC 0/1	0	3	3	
Total	14	3	19	

^a^Fisher’s exact test.

**Table 4:** Association of HER2 immunocytochemistry with HER2 gene amplification in dogs with urothelial carcinoma.

HER2 gene amplification	HER2 immunocytochemistry			*P*^a^
	ICC 2/3	ICC 0/1	Total	
Amplified	11	1	12	0.04
Non-amplified	18	14	32	
Total	29	15	44	

^a^Fisher’s exact test.

A somatic point mutation in the BRAF gene (BRAF^V595E^), which is homologous to the human BRAF^V600E^ mutation, is present in over 70% of dogs with urothelial carcinoma^26^. Of the 44 dogs with lapatinib/piroxicam, BRAF^V595E^ mutation was detected in 32 (73%) cases (Fig. S1a and Table S1). There was no association between BRAF^V595E^ mutation and clinical response in dogs treated with lapatinib/piroxicam (Fig. S1b). PFS and OS were not related to the BRAF^V595E^ mutation (Fig. S1c).

## Discussion

In the present study, we showed that first-line lapatinib in combination with piroxicam was associated with clinical benefit in a canine muscle-invasive urothelial carcinoma model, especially in HER2 positive tumor. The objective response rates (ORRs) of lapatinib/piroxicam treatment were 55% (24 of 44 dogs), and the median PFS and OS were 193 and 435 days, respectively. Although a randomized clinical trial would be needed to the establish the significance of difference, the results compare well with those of first-line cisplatin-based regimens (ORRs 11–73%; median PFS 78–186 days; median OS 179–329 days) or mitoxantrone in combination with piroxicam (ORRs 35%; median PFS 194 days; median OS 350 days) in dogs with urothelial carcinoma^27–30^. The reported median OS in dogs with urothelial carcinoma following surgery and adjuvant therapy ranges from 348 to 385 days^31,32^. To our knowledge, this study is the first clinical trial to report an EGFR/HER2 inhibitor as a first-line treatment in dogs with urothelial carcinoma.

Lapatinib treatment was well tolerated by dogs. Most treatment-related adverse events were grade 1 or 2. Elevated liver enzymes, vomiting, and diarrhea were manageable with hepatoprotective, antiemetic, and antidiarrheal agents, respectively. Dermatologic adverse events, such as hyperpigmentation, pruritus, and alopecia, were observed in mild conditions and intervention was not required in most cases. Skin ulceration observed in one dog improved with antibacterial ointment. Severe pulmonary hypertension with syncope (maximal tricuspid regurgitation velocity, 4.6 m/s; estimated systolic pulmonary artery pressure, 86 mmHg) was observed in one case, although it is unclear whether this was an adverse event of lapatinib. In this case, pulmonary hypertension and syncope were improved with sildenafil (maximal tricuspid regurgitation velocity, 3.9 m/s; estimated systolic pulmonary artery pressure, 60 mmHg). The rate of increased creatinine in dogs with lapatinib/piroxicam and piroxicam alone was similar in this trial, indicating that renal toxicity was mainly induced by piroxicam rather than lapatinib. In humans, the most common adverse events associated with lapatinib treatment were diarrhea (39–67%), skin rash (5–46%), fatigue (10–39%), nausea (24–27%), and vomiting (19–22%), which are manageable and reversible^10,11^. These safety data, including a lack of renal toxicity, suggest that patients with urothelial cancer, who are often elderly and predisposed to having renal impairment, may be better able to tolerate lapatinib treatment than chemotherapy.

This study demonstrated a greater clinical response to lapatinib treatment and significant prolongation of PFS and OS in dogs with HER2 overexpression than in those with low HER2 expression. HER2 gene amplification was associated with longer OS but not PFS and clinical response to lapatinib. These findings may be explained by the lower prevalence of HER2 gene amplification than HER2 overexpression. HER2 gene amplification was detected in only 38% of dogs with HER2 overexpression, indicating other mechanisms underlying HER2 overexpression. Further studies are necessary to investigate the detailed mechanisms of HER2 overexpression in canine urothelial carcinoma. Given that more than 60% of canine urothelial carcinomas harbor HER2 overexpression, the companion dog model could be a highly relevant platform for comparative cancer research to understand the molecular events leading to the HER2 pathway and further develop a HER2-targeted therapy.

Subset analysis of canine urothelial carcinoma showed that the BRAF^V595E^ mutation is not prognostic in dogs treated with lapatinib/piroxicam. Similar results of no association between BRAF^V595E^ mutation and outcomes have been reported in dogs with urothelial carcinoma^32,33^. Although the impact of BRAF^V595E^ mutations on canine urothelial carcinoma remains unclear, recent studies have reported the involvement of molecular pathogenesis. Parker et al. have investigated differentially expressed genes and pathways in canine urothelial carcinoma with or without BRAF^V595E^ mutation, identifying that tissue development, cell cycle, and cell death pathways are enriched in BRAF^V595E^ mutant tumors, while the immune response genes are upregulated in BRAF wild-type tumors^33^. Yoshitake et al. demonstrated that BRAF^V595E^ mutation accelerates an immunosuppressive lipid mediator prostaglandin E_2_ production in canine urothelial carcinoma cells^34^. Our previous studies showed that regulatory T cell recruitment in canine urothelial carcinoma is caused by BRAF^V595E^ mutation-driven CCL17/CCR4 pathway^35,36^. These results suggest that BRAF^V595E^ mutation induces immunosuppressive microenvironment in canine urothelial carcinoma. More extensive studies are needed to examine the association between BRAF^V595E^ mutation and prognosis in dogs with urothelial carcinoma.

This study has several limitations. First, this canine clinical trial was a prospective non-randomized clinical trial but not a randomized design. Randomized controlled trials are needed to prove the efficacy of lapatinib in canine urothelial carcinoma. Second, tumor samples were not evaluated in full thickness pathology because most of the cases were biopsied by cystoscopy or catheter aspiration. Therefore, histological grading based on previous reports on canine proliferative urothelial lesions^37,38^ could not be applied. Further study is needed to investigate the association between pathological features and treatment response. Third, HER2 overexpression in all cases was evaluated using ICC, but not IHC. Biopsy samples of tumor tissue were too small to be used for HER2 immunostaining because they were preferentially used for pathology. However, both ICC and IHC were performed in 19 dogs with urothelial carcinoma and showed a significant correlation, suggesting the availability of ICC for the evaluation of HER2 expression. Fourth, HER2 status was evaluated only in cases with lapatinib/piroxicam but not in cases with piroxicam alone. There is a potential risk that the prevalence of HER2 overexpression or gene amplification may differ between treatment groups, which may affect survival. Fifth, this study did not evaluate the expression and gene amplification of EGFR, another target of lapatinib. Our previous study showed that the antitumor effect of lapatinib in canine urothelial carcinoma cell lines is associated with HER2 expression, but not with EGFR expression^23^, therefore we evaluated HER2 status as a biomarker for response to lapatinib in this canine trial. However, this trial included some dogs that did not overexpress HER2 and responded to lapatinib. Therefore, it is necessary to investigate the association between EGFR expression and the therapeutic effects of lapatinib. In addition, we plan to design clinical trials in which EGFR and/or HER2 positive cases are selected for inclusion.

Over the last few decades, human tumor cell line-based or patient-derived xenograft models have been applied to preclinical testing of anti-cancer agents. However, these immunocompromised mouse models do not accurately predict the outcome of drugs in humans^39^. Current models for urothelial carcinoma include carcinogen-induced, engraftment, and genetically engineered mouse models. Although these mouse models are essential for bladder cancer research, few models develop muscle-invasive or metastatic phenotypes and possess cancer heterogeneity^40^. There is compelling evidence that dogs with spontaneous urothelial carcinoma better resemble humans with regard to heterogeneity, clinical sign, histopathology, disease progression, metastatic behavior, and immunologic phenotypes and can serve as a highly relevant model for human muscle-invasive bladder cancer to complement other animal models^17,33,41^. Comparative clinical trials conducted in companion dogs play an important and growing role in cancer research and drug development efforts. A variety of emerging therapies, such as cyclooxygenase inhibitors, demethylating agents, and folate analogs, have demonstrated clinical activity for the treatment of muscle-invasive urothelial carcinoma in dogs^41–44^, thereby accelerating human clinical trials. Expanding the application of the canine urothelial carcinoma model is expected to improve the outcome of urinary bladder cancer in humans and dogs.

In conclusion, lapatinib in combination with piroxicam is an encouraging option as a first-line treatment for dogs with muscle-invasive urothelial carcinoma with a low incidence of clinically relevant toxicities. This canine model of muscle-invasive urothelial carcinoma also paves the way for the translation of first-line lapatinib therapy to human patients with muscle-invasive bladder cancer.

## Methods

### Ethical statements

As a prospective, single-center, open-label, non-randomized clinical trial comparing dogs with urothelial carcinoma treated with either piroxicam/lapatinib or piroxicam alone, the Animal Care and Clinical Research Committees of the VMC-UT approved this study (approval no. VMC2017-01). Written informed consent was obtained from all the dog owners. All experimental methods were performed in accordance with ARRIVE guidelines. All methods were carried out in accordance with relevant guidelines and regulations.

### Canine clinical trial design and interventions

A phase II canine clinical trial was conducted at the VMC-UT. Client-owned pet dogs with histologically confirmed muscle-invasive urothelial carcinoma (transitional cell carcinoma) were enrolled in a canine clinical trial of lapatinib, excluding dogs treated with chemotherapy or radiotherapy from this clinical trial. All dogs underwent a physical examination, laboratory evaluations (complete blood counts, serum biochemical profile, and urinalysis), 3-view thoracic radiography, 2-view abdominal radiography, and abdominal ultrasonography as part of the eligibility screening within 7 d prior to study enrollment. Dogs that had any significant comorbid illnesses were excluded. In addition, dogs with any of the following criteria were considered ineligible for enrollment: hydronephrosis (obstruction of the ureter by tumor invasion), creatinine > 3.0 mg/dL, total bilirubin > 2.0 mg/dL, hematocrit < 25%, or platelets < 50,000/mL. Before treatment, the tumor stage was defined according to the WHO criteria for canine bladder cancer^24,25^. In the lapatinib/piroxicam arm, lapatinib (Tykerb^®^, 20–30 mg/kg; Novartis, Basel, Switzerland) in combination with piroxicam (0.3 mg/kg; Pfizer, New York City, NY, USA) was orally administered to dogs with urothelial carcinoma once every 24 h. The dosage and administration interval were based on a previous study^45^. In the piroxicam arm, only piroxicam was orally administered to dogs with muscle-invasive urothelial carcinoma once every 24 h. Treatment was continued until dogs experienced disease progression, had unacceptable toxicity, or their owners stopped adhering to the study protocol. No placebo control, blinding, or randomization was performed in this study.

### Clinical assessment

Dogs were examined for clinical responses and toxicity at least once every 4 weeks via owner observations, physical examination, complete blood counts, serum chemical profiles, 3-view thoracic radiography, and abdominal ultrasonography. A single ultrasound operator measured bladder and/or urethra masses following a standardized mapping procedure and recorded the estimated tumor volume^46,47^. We used ultrasound for imaging as it could be conducted without general anesthesia (as would be required for computed tomography in dogs). The tumor response was defined as follows: CR; no tumor lesions detected, PR; ≥ 50% decrease in tumor volume and no new tumor lesions, SD; < 50% change in tumor volume and no new tumor lesions, and PD; ≥ 50% increase in tumor volume or the development of new tumor lesions^42,43^. PFS was defined as the time from the start of treatment until PD or death at the end of the study (July 1, 2021), and OS as the time from the start of treatment until death of the animal at the end of the study. Adverse events were assessed using the Veterinary Cooperative Oncology Group (VCOG) criteria^48^. Azotemia in adverse events was defined as azotemia without tumor-induced hydronephrosis.

### Immunohistochemistry

HER2 expression was examined in the tumor tissue by IHC^19,49^. Briefly, 4-μm sections were deparaffinized, rehydrated, and treated with 3% hydrogen peroxide-methanol at room temperature for 5 min and then heated in a water bath at 98 °C for 40 min in the target retrieval solution (pH 9.0; Dako, Glostrup, Denmark). The sections were blocked with 8% skim milk in Tris-buffered saline (TBS) at 37 °C for 40 min and then incubated with a rabbit polyclonal anti-human c-erbB-2 oncoprotein (HER2/neu, 1:100 dilution; Dako) at 37 °C for 40 min. The sections were incubated with the EnVision polymer reagent for rabbit IgG (Dako) at 37 °C for 60 min. Then, the reaction products were visualized using 3,3-diaminobenzidine. HER2 immunoreactivity was quantified based on a previous study^19^. Samples with HER2 IHC scores of 0 and 1 were classified as negative, and those with scores of 2 and 3 were classified as positive.

### Immunocytochemistry

HER2 expression was examined in urine sediments using ICC. Tumor cells in the urine sediments were collected by aspiration biopsy with a urinary catheter from dogs that received the lapatinib treatment. Samples were centrifuged at 400×*g* for 5 min, and the supernatant was discarded. The cells were washed in Hanks’ Balanced Salt Solution (HBSS; Thermo Fisher Scientific, Waltham, MA, USA) and erythrocytes were removed from the samples using the hemolytic ACK buffer (Thermo Fisher Scientific) at room temperature for 5 min. After washing with HBSS, cytological smears were prepared, thoroughly air-dried for 20 min, and then stored at − 30 °C until later use. The slides were fixed with ice-cold methanol and blocked with 5% skim milk in TBS at room temperature for 10 min. The samples were incubated with a rabbit polyclonal anti-human c-erbB-2 oncoprotein (1:100 dilution; Dako) at 37 °C for 15 min and then with the EnVision polymer reagent for rabbit IgG (Dako) at 37 °C for 10 min. The reaction products were visualized with diaminobenzidine, and counterstaining was conducted with Mayer’s hematoxylin. As shown in Fig. 2a, a score of 0 denotes no reactivity at all, a score of 1 + represents incomplete and weak immunoreactivity in < 10% of tumor cells, a score of 2 + represents incomplete but intense immunoreactivity in ≥ 10% of tumor cells, and a score of 3 + represents intense with complete immunoreactivity (known as “chicken-wire pattern”) in ≥ 10% of tumor cells. Samples with scores of 0 and 1 + were classified as HER2-negative, and those with scores of 2 + and 3 + as HER2-positive.

### Biomarkers

For biomarker assessment, we collected fresh urine samples before treatment for HER2 overexpression, HER2 gene amplification, and BRAF^V595E^ mutation. HER2 expression was assessed using the ICC, as described above. HER2 gene amplification and BRAF^V595E^ mutation were examined by digital PCR assay using genomic DNA isolated from urine sediments, as previously described^36,49^.

### Endpoints and statistical analyses

The primary endpoint of this study was the PFS. The secondary endpoints were OS, response rates, adverse events, and outcome of subsets depending on the biomarker status (HER2 overexpression, HER2 gene amplification, or BRAF^V595E^ mutation). A power analysis was performed to estimate the number of dogs needed to detect a statistically significant difference in PFS between the 2 treatment groups using the Power and Sample Size Calculation software v.3.1.6 (https://biostat.app.vumc.org/wiki/Main/PowerSampleSize)^50^. Based on the literature, the duration of PFS in dogs treated with piroxicam alone was assumed to be 120 days^14^. A minimum of 76 dogs were required for enrollment on the basis of an alpha level of 0.05 to detect a twofold longer median PFS in the lapatinib group with 80% power, although there was a lack of previous data to guide this estimation. The Cochran–Armitage test for trend was used to evaluate the association of clinical response with treatment, HER2 ICC score, HER2 gene amplification, or the BRAF^V595E^ mutation. Fisher’s exact test was used to compare adverse events between the treatment groups. The Mann–Whitney *U* test was used to compare continuous variables between the treatment groups. Fisher’s exact test was used to compare categorical variables between the treatment groups and determine the association between HER2 ICC score and HER2 IHC score or HER2 gene amplification. Survival curves were generated using the Kaplan–Meier method. For PFS and OS, *P* values were calculated using the log-rank test. Hazard ratios and 95% confidence intervals (CIs) were calculated using the Cox proportional hazard model. We used JMP Pro v.11.2 (SAS Institute, Cary, NC, USA) for statistical analyses. Statistical significance was set at *P* < 0.05.

## Supplementary Information

Supplementary Information.
