# Supplementary material for: Lapatinib as first-line treatment for muscle-invasive urothelial carcinoma in dogs
Source: Sci Rep. 2022 Jan 13;12:4. doi: 10.1038/s41598-021-04229-0 (PMC8758709; doi:10.1038/s41598-021-04229-0)
Supplement: Supplementary file 1 — Supplementary Information. [file 41598_2021_4229_MOESM1_ESM.pdf]

Supplementary Materials for

Lapatinib as first-line treatment for muscle-invasive urothelial carcinoma in dogs

Shingo Maeda*, Kosei Sakai, Kenjiro Kaji, Aki Iio, Maho Nakazawa, Tomoki Motegi,

Tomohiro Yonezawa, Yasuyuki Momoi

*Corresponding author. E-mail: amaeda@g.ecc.u-tokyo.ac.jp

The PDF file includes:

Fig. S1. $BRAF^{V595E}$ mutation is not associated with clinical responses and survival in dogs with urothelial carcinoma.

Table S1. Characteristics of dogs with urothelial carcinoma in the clinical trial.

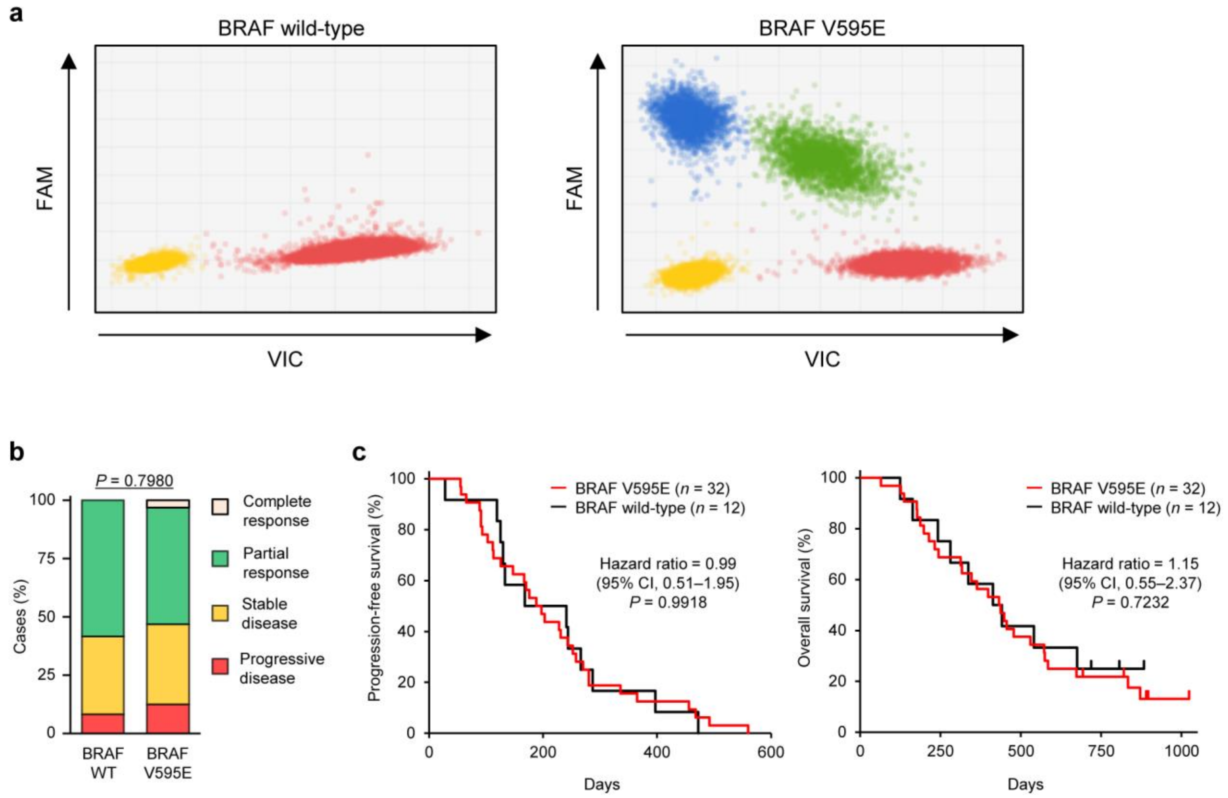

Figure S1. BRAF^{V595E} mutation is not associated with clinical responses and survival in dogs with urothelial carcinoma. (**a**) Digital PCR assay for genotyping of BRAF in dogs with urothelial carcinoma. X-axis: VIC, wild-type BRAF. Y-axis: FAM, BRAF^{V595E} mutation. Absence of blue and green clusters indicates urothelial carcinoma with wild-type BRAF (left). Presence of blue and green clusters indicates urothelial carcinoma with BRAF^{V595E} mutation (right). (**b**) Clinical responses in canine urothelial carcinoma cases with wild-type (WT) BRAF (n = 12) or BRAF^{V595E} mutation (n = 32). Cochran–Armitage test. (**c**) Progression-free survival (left) and overall survival (right) in canine urothelial carcinoma cases with wild-type (WT) BRAF (n = 12, black line) or BRAF^{V595E} mutation (n = 32, red line). Log-rank test.

Table S1. Characteristics of dogs with urothelial carcinoma in the clinical trial.

Case ID	Age (years)	Sex[a]	Breed	TNM classification	$BRAF^{V595E}$ mutation	HER2 ICC[b] score	HER2 IHC[c] score	HER2 gene amplification	Treatment	Response[d]	PFS[e] (days)	OS[f] (days)
L1	11.2	FN	English Setter	T2N1M0	Wild-type	2	Not tested	Yes	Lapatinib/piroxicam	SD	133	413
L2	12.3	FN	Miniature Dachshund	T2N0M0	Mutation	1	3	No	Lapatinib/piroxicam	PR	203	313
L3	16.2	FN	West Highland White Terrier	T2N0M0	Mutation	1	Not tested	No	Lapatinib/piroxicam	PD	89	363
L4	12.3	FN	Italian Greyhound	T2N0M0	Mutation	2	Not tested	No	Lapatinib/piroxicam	PR	560	584
L5	16.0	MI	Toy Poodle	T2N0M0	Wild-type	0	Not tested	No	Lapatinib/piroxicam	SD	125	337
L6	11.8	MN	Scottish Terrier	T3N1M0	Mutation	2	Not tested	Yes	Lapatinib/piroxicam	PR	231	347
L7	10.0	FN	Shetland Sheepdog	T2N1M1	Wild-type	1	Not tested	No	Lapatinib/piroxicam	PD	28	125
L8	13.2	FN	Yorkshire Terrier	T2N0M0	Wild-type	2	3	Yes	Lapatinib/piroxicam	SD	397	675
L9	11.5	FN	Miniature Dachshund	T2N1M1	Mutation	2	Not tested	No	Lapatinib/piroxicam	PR	103	214
L10	13.8	FN	Miniature Dachshund	T2N1M0	Wild-type	2	Not tested	No	Lapatinib/piroxicam	PR	287	441
L11	11.1	FN	Pomeranian	T2N0M0	Mutation	3	Not tested	Yes	Lapatinib/piroxicam	PR	365	833
L12	12.3	FN	Miniature Schnauzer	T3N1M0	Mutation	2	Not tested	No	Lapatinib/piroxicam	PR	91	176
L13	9.3	FN	Miniature Dachshund	T2N0M0	Mutation	1	Not tested	No	Lapatinib/piroxicam	PD	56	198
L14	9.0	FI	Papillon	T2N0M0	Mutation	1	Not tested	No	Lapatinib/piroxicam	PR	468	871
L15	10.8	FN	Chihuahua	T2N0M0	Mutation	3	Not tested	No	Lapatinib/piroxicam	PR	243	432
L16	12.8	MN	Chihuahua	T3N1M0	Mutation	1	Not tested	No	Lapatinib/piroxicam	SD	112	127
L17	13.8	FN	Chihuahua	T2N0M0	Mutation	3	Not tested	Yes	Lapatinib/piroxicam	CR	456	456
L18	9.0	MN	Mongrel	T3N1M0	Wild-type	3	Not tested	No	Lapatinib/piroxicam	SD	119	242
L19	14.9	MI	Toy Poodle	T2N0M0	Mutation	2	Not tested	Yes	Lapatinib/piroxicam	PR	280	1023
L20	11.5	FN	Miniature Dachshund	T2N0M0	Mutation	3	2	No	Lapatinib/piroxicam	SD	188	673
L21	14.5	FN	Mongrel	T2N1M0	Mutation	1	Not tested	Yes	Lapatinib/piroxicam	SD	126	437
L22	10.8	FN	Pembroke Welsh Corgi	T2N0M0	Mutation	3	2	Yes	Lapatinib/piroxicam	PD	55	890
L23	14.4	MI	Jack Russell Terrier	T2N1M0	Mutation	2	2	No	Lapatinib/piroxicam	PR	170	530
L24	11.2	MN	Shetland Sheepdog	T2N0M0	Mutation	2	2	Yes	Lapatinib/piroxicam	PR	252	896
L25	10.8	FI	Shetland Sheepdog	T2N0M0	Mutation	3	2	No	Lapatinib/piroxicam	SD	176	398
L26	13.8	FN	Pomeranian	T2N0M0	Mutation	3	Not tested	No	Lapatinib/piroxicam	PR	280	820
L27	14.3	MN	Mongrel	T2N0M0	Wild-type	3	3	No	Lapatinib/piroxicam	PR	266	883
L28	12.9	FN	Jack Russell Terrier	T2N0M0	Wild-type	3	2	No	Lapatinib/piroxicam	PR	244	541
L29	9.9	FN	Bernese Mountain Dog	T2N1M0	Mutation	0	1	No	Lapatinib/piroxicam	SD	197	244
L30	13.3	FI	Chihuahua	T2N1M0	Mutation	1	1	No	Lapatinib/piroxicam	SD	167	177
L31	10.8	MN	Chihuahua	T2N0M0	Wild-type	1	1	No	Lapatinib/piroxicam	PR	472	806
L32	13.5	FN	Mongrel	T2N0M0	Mutation	2	2	No	Lapatinib/piroxicam	SD	147	317
L33	13.5	MN	Pembroke Welsh Corgi	T2N0M0	Wild-type	1	2	No	Lapatinib/piroxicam	PR	130	163
L34	16.0	FN	Miniature Dachshund	T2N0M0	Mutation	1	Not tested	No	Lapatinib/piroxicam	PR	65	65
L35	9.1	MI	Shetland Sheepdog	T2N0M0	Mutation	2	2	No	Lapatinib/piroxicam	PR	228	232
L36	14.1	MN	Miniature Pinscher	T2N0M0	Mutation	2	2	Yes	Lapatinib/piroxicam	SD	336	575
L37	13.3	FN	Jack Russell Terrier	T2N0M0	Wild-type	2	2	No	Lapatinib/piroxicam	PR	241	281
L38	10.3	FN	Maltese	T2N0M0	Mutation	2	2	No	Lapatinib/piroxicam	PR	270	448
L39	9.3	FN	Toy Poodle	T3N0M0	Mutation	2	Not tested	No	Lapatinib/piroxicam	SD	492	573
L40	13.5	FN	Labrador Retriever	T2N0M0	Mutation	2	Not tested	No	Lapatinib/piroxicam	PR	113	478
L41	11.5	FI	Shetland Sheepdog	T2N0M0	Mutation	1	Not tested	No	Lapatinib/piroxicam	SD	93	137
L42	6.3	FI	Norwich Terrier	T2N1M0	Mutation	0	Not tested	No	Lapatinib/piroxicam	PD	91	187
L43	13.1	FN	Miniature Dachshund	T2N0M0	Wild-type	2	2	Yes	Lapatinib/piroxicam	PR	168	719
L44	10.2	FN	Wire Fox Terrier	T2N0M0	Mutation	2	Not tested	Yes	Lapatinib/piroxicam	SD	258	693
P1	13.5	MI	Pembroke Welsh Corgi	T2N0M0	Wild-type	Not tested	Not tested	Not tested	Piroxicam	SD	68	528
P2	12.6	MN	Mongrel	T2N1M0	Mutation	Not tested	Not tested	Not tested	Piroxicam	PD	60	225
P3	9.2	MN	Chihuahua	T2N0M0	Wild-type	Not tested	Not tested	Not tested	Piroxicam	SD	108	166
P4	11.1	FN	Pembroke Welsh Corgi	T2N1M0	Mutation	Not tested	Not tested	Not tested	Piroxicam	SD	98	113
P5	15.9	FI	West Highland White Terrier	T3N1M0	Wild-type	Not tested	Not tested	Not tested	Piroxicam	SD	57	129
P6	12.7	FN	Toy Poodle	T2N0M0	Mutation	Not tested	Not tested	Not tested	Piroxicam	PD	34	132
P7	14.5	FN	Labrador Retriever	T3N0M0	Mutation	Not tested	Not tested	Not tested	Piroxicam	PD	43	43
P8	11.8	FN	French Bulldog	T2N0M0	Mutation	Not tested	Not tested	Not tested	Piroxicam	SD	54	265
P9	10.9	MI	Yorkshire Terrier	T2N0M0	Mutation	Not tested	Not tested	Not tested	Piroxicam	SD	161	729
P10	9.1	FN	Shetland Sheepdog	T2N0M0	Mutation	Not tested	Not tested	Not tested	Piroxicam	SD	163	223
P11	11.6	FI	Papillon	T2N0M0	Wild-type	Not tested	Not tested	Not tested	Piroxicam	SD	84	340
P12	13.4	FN	Miniature Dachshund	T2N1M0	Mutation	Not tested	Not tested	Not tested	Piroxicam	SD	42	486
P13	14.2	FN	Pembroke Welsh Corgi	T2N0M0	Mutation	Not tested	Not tested	Not tested	Piroxicam	SD	68	108
P14	9.5	FI	Maltese	T2N0M0	Mutation	Not tested	Not tested	Not tested	Piroxicam	SD	153	210
P15	13.5	FN	Toy Poodle	T2N0M0	Mutation	Not tested	Not tested	Not tested	Piroxicam	PR	139	613
P16	9.8	FI	Border Collie	T2N0M0	Wild-type	Not tested	Not tested	Not tested	Piroxicam	PR	126	130
P17	11.9	FN	Pomeranian	T2N0M0	Mutation	Not tested	Not tested	Not tested	Piroxicam	PD	21	138
P18	12.8	FN	Miniature Dachshund	T2N0M0	Mutation	Not tested	Not tested	Not tested	Piroxicam	SD	95	361
P19	8.9	FN	Chihuahua	T2N0M1	Mutation	Not tested	Not tested	Not tested	Piroxicam	SD	56	114
P20	14.9	MI	Toy Poodle	T2N0M0	Wild-type	Not tested	Not tested	Not tested	Piroxicam	SD	122	516
P21	7	FI	Miniature Dachshund	T2N0M0	Mutation	Not tested	Not tested	Not tested	Piroxicam	PD	33	228
P22	9.1	MN	Shetland Sheepdog	T2N1M0	Mutation	Not tested	Not tested	Not tested	Piroxicam	SD	157	222
P23	13.9	FN	Mongrel	T3N1M1	Mutation	Not tested	Not tested	Not tested	Piroxicam	SD	55	55
P24	11.1	FN	Mongrel	T2N1M0	Mutation	Not tested	Not tested	Not tested	Piroxicam	SD	113	113
P25	9.1	MI	Scottish Terrier	T2N0M0	Mutation	Not tested	Not tested	Not tested	Piroxicam	PR	168	367
P26	13.6	MN	Labrador Retriever	T2N0M0	Wild-type	Not tested	Not tested	Not tested	Piroxicam	PD	70	164
P27	9.6	FN	Scottish Terrier	T2N0M0	Mutation	Not tested	Not tested	Not tested	Piroxicam	SD	46	190
P28	11.9	FN	Miniature Schnauzer	T2N0M0	Mutation	Not tested	Not tested	Not tested	Piroxicam	SD	113	267
P29	11.9	FN	Miniature Dachshund	T2N0M0	Mutation	Not tested	Not tested	Not tested	Piroxicam	PD	41	41
P30	11.5	MN	Miniature Pinscher	T2N0M0	Mutation	Not tested	Not tested	Not tested	Piroxicam	SD	83	140
P31	11.9	FN	Mongrel	T2N0M0	Mutation	Not tested	Not tested	Not tested	Piroxicam	SD	96	170
P32	11.5	FN	Miniature Dachshund	T3N0M0	Mutation	Not tested	Not tested	Not tested	Piroxicam	SD	98	308
P33	14.9	FN	Miniature Dachshund	T2N0M0	Wild-type	Not tested	Not tested	Not tested	Piroxicam	SD	104	129
P34	12.3	FI	Shih Tzu	T2N0M1	Mutation	Not tested	Not tested	Not tested	Piroxicam	PD	40	51
P35	7.5	MN	Chihuahua	T2N0M0	Wild-type	Not tested	Not tested	Not tested	Piroxicam	SD	43	619
P36	10.7	MN	Papillon	T2N0M0	Mutation	Not tested	Not tested	Not tested	Piroxicam	PR	318	534
P37	11.8	FN	Border Collie	T2N1M0	Wild-type	Not tested	Not tested	Not tested	Piroxicam	PD	55	74
P38	15.3	MN	West Highland White Terrier	T2N1M0	Mutation	Not tested	Not tested	Not tested	Piroxicam	PD	33	71
P39	10.9	MI	Bernese Mountain Dog	T2N0M0	Mutation	Not tested	Not tested	Not tested	Piroxicam	SD	149	245
P40	12.5	FN	Chihuahua	T2N0M0	Mutation	Not tested	Not tested	Not tested	Piroxicam	SD	182	725
P41	12.3	MN	Shiba Inu	T2N0M0	Wild-type	Not tested	Not tested	Not tested	Piroxicam	SD	203	434
P42	10.6	MN	Cavalier King Charles Spaniel	T2N1M0	Mutation	Not tested	Not tested	Not tested	Piroxicam	SD	245	322

[a]FN, female neutered; FI, female intact; MN, male neutered; MI, male intact. [b]ICC, immunocytochemistry. [c]IHC, immunohistochemistry. [d]CR, complete remission; PR, partial response; SD, stable disease; PD, progressive disease. [e]PFS, progression-free survival. [f]OS, overall survival.
